# Expression Patterns of Ezrin and AJAP1 and Clinical Significance in Breast Cancer

**DOI:** 10.3389/fonc.2022.831507

**Published:** 2022-03-04

**Authors:** Cong Xu, Feng Wang, Li Hao, Jing Liu, Benjie Shan, Shuhua Lv, Xinghua Han, Yueyin Pan, Yun Niu

**Affiliations:** ^1^ Department of Medical Oncology, The First Affiliated Hospital of University of Science and Technology of China (USTC), Division of Life Sciences and Medicine, University of Science and Technology of China, Hefei, China; ^2^ Tianjin Medical University Cancer Institute and Hospital, National Clinical Research Center of Cancer, Key Laboratory of Cancer Prevention and Therapy, Tianjin’s Clinical Research Center for Cancer, Tianjin, China; ^3^ Key Laboratory of Breast Cancer Prevention and Therapy, Tianjin Medical University, Ministry of Education, Tianjin, China; ^4^ Department of Breast Cancer Pathology and Research Laboratory, Tianjin Medical University Cancer Institute and Hospital, Tianjin, China; ^5^ Department of Pathology, Tianjin Union Medical Center, Tianjin People’s Hospital, Tianjin, China

**Keywords:** AJAP1, Ezrin, adherens junction-associated 1, shrew-1, prognosis

## Abstract

Ezrin and adherens junction-associated protein 1 (AJAP1) are structural proteins which are involved in numerous human malignancies. However, little is known about the relationship between them in breast cancer. This study was set out to investigate the relationship between them and to further explore the mechanism of AJAP1-mediating cytoskeleton in breast cancer progression. Ezrin and AJAP1 expressions were detected in 377 samples of breast cancer by immunohistochemistry, and different expression patterns between AJAP1 and Ezrin with clinicopathological parameters were analyzed. Besides, univariate and multivariate Cox models were used to evaluate their prognostic potential. Enzyme-linked immunosorbent assay, Western blot, qRT-PCR, and phalloidin staining of F-actin were used to explore the relationship and the mechanism between AJAP1 and Ezrin in cytoskeleton arrangement. 377 cases of breast cancer results showed that AJAP1 expression was negatively related with histological grade and lymph node involvement and could be an independent prognosis marker of breast cancer. AJAP1 expression tended to be higher in the Ezrin-negative expression case. Patients with AJAP1^negative^ and Ezrin^positive^ expression had a worse prognosis (*p* < 0.0001) and shorter DFS (*p* = 0.015). More importantly, AJAP1 depletion increased the cell ability of F-actin formation through promoting Ezrin expression. AJAP1 depletion might mediate breast cancer malignancy potential through promoting Ezrin expression and cytoskeleton formation.

## Introduction

Breast cancer, the leading cancer killer across the world, has been threatening women’s health and its morbidity and mortality have increased recently (1). Tumor invasion and metastasis are two important reasons resulting in breast cancer development. Besides, cytoskeleton-associated proteins also play key roles in this process (2).

Adherens junction-associated protein 1 (AJAP1) is also named shrew-1; it was firstly found as a novel transmembrane protein of adherens junctions in epithelial cells (3, 4). AJAP1 has been proved as a tumor suppressor in glioma (5–7), hepatocellular carcinoma (8, 9), esophagus carcinoma (10), oligodendroglioma (11), and endometrial cancer (12). Especially in glioblastoma, AJAP1’s role has been fully explored. For example, both Han et al. (7) and Yang et al. (5) testified that AJAP1 expression affected the cytoskeleton in glioblastoma and predicted poor prognosis. AJAP1 also participated in many transduction signals of cell–cell and cell–extracellular matrix related to cell motility, migration, and invasion ability. Our previous study verified that AJAP1 depletion promoted breast cancer progression by accelerating β-catenin nuclear transaction (13). However, data about the breast cancer are still scarce.

Ezrin is an important member of ERM (ezrin, radixin, and moesin) proteins, which is critical for structural stability and integrity maintenance (14). Recent studies show that Ezrin can act as a tumor metastasis regulator in invasion and metastasis of many types of cancer (15–18). Besides, it also mediates many cellular activities such as polarity, motility, adhesion, and survival which are associated with cancer development and progression (19, 20). Overexpression of Ezrin is seen as a tumor prognosis marker of several human cancers (21–26). In breast cancer, Ezrin also plays an instrumental role in mediating tumor progression and metastasis (27). However, more data on the mechanism of Ezrin in breast cancer need to be further explored.

The above data showed that both adherens junction-associated protein 1 (AJAP1) and Ezrin were structural proteins. In the current study, we first investigated the relationship between Ezrin and AJAP1 expression and then evaluated their prognosis accuracy in predicting prognosis of breast cancer patients. More importantly, these results might bring a new insight on the feedback loop of AJAP1 and Ezrin in breast cancer progression.

## Materials and Methods

### Patients’ Selection and Related Information

377 patients of breast cancer who underwent mastectomy and a diagnosis of invasive ductal carcinoma were made based on a histopathological evaluation between 2005 and 2006 at Tianjin Medical University Cancer Institute and Hospital. They were randomly selected, and all were informed with study information. None of them received preoperative treatment such as chemotherapy and radiotherapy. Besides, their clinicopathologic data were available. Patients of this cohort were female, and the age range is 27 to 82 years (median age is 51 years).

### Immunohistochemistry and Evaluation

Immunohistochemistry assay was carried out as in our previous study (13). All primary antibodies included ER (ZETA, SP1; 1:200 dilution), PR (ZETA, SP2; 1:200 dilution), epidermal growth factor receptor 2 (HER2) (Invitrogen, Carlsbad, CA, USA, CB11; 1:100 dilution), Ki67 (Invitrogen, K-2; 1:100 dilution), AJAP1 (Abcam, Cambridge, MA, USA, ab205496, 1:100 dilution), and Ezrin (Santa Cruz Biotechnology, Dallas, TX, USA, sc-58758;1:200 dilution), respectively. Sections of normal breast tissue were processed simultaneously and served as positive controls for ER and PR. Similarly, HER2- and Ki67-positive breast cancer tissues were used as positive controls for HER2 and Ki67, respectively. Besides,AJAP1-positive glioma tissues represent AJAP1 positive, Ezrin-positive breast cancer tissues represent Ezrin positive. In addition, normal goat serum substituted primary antibodies as negative controls. Besides, we have also used positive and negative controls for each run. The AJAP1 and Ezrin score evaluation was based on the location of immunoreactivity, the percentage of stained cells. The percentage of positivity of the tumor was scored as “0” (no tumor cells), “1” (1%–25%), “2” (26%–50%), “3” (51%–75%), and “4” (75%–100%). The staining intensity of the positive tumor cells was scored as “0” (no staining), “1” (weak staining), “2” (moderate staining), and “3” (strong staining). Eventually, the multiplier of scores is as follows: 0–3 for negative expression, 4–12 for positive expression.

### Follow-Up

All the patients had decent follow-up data which were obtained by medical records or telephone calls. The time of last follow-up was August 1, 2018. Follow-up time ranges from 85 to 144 months (average 104 months).

### Cell Culture

T47D and MDA-MB-231 cell lines were obtained from the American Type Culture Collection (ATCC, Manassas, USA) for further study. They were cultured in RPMI-1640 medium (Gibco, Grand Island, NY, USA) with 10% FBS and 1% penicillin/streptomycin (HyClone, Logan, UT, USA) in a 5% CO_2_ incubator at 37°C.

### Western Blot

Total proteins were extracted by using RIPA with PMSF according to the manufacturer’s protocol. An equal amount (30 µg) of samples was separated on 10% SDS-PAGE gels and transferred to PVDF membranes (Millipore, Burlington, MA, USA). Then, the membranes were blocked in 5% non-fat milk for 1 h. Eventually, these membranes were detected using the ECL detection Kit (Solarbio, Beijing, China) after incubating the primary antibodies including anti-AJAP1 (AJAP1; Abcam; ab205496, rabbit secondary antibody) and anti-Ezrin (Ezrin; Santa Cruz Biotechnology, sc-58758, mouse secondary antibody).

### Cell Transfection and Plasmids

The AJAP1 siRNAs and control plasmids are shown as in our previous study (13). T47D and MDA-MB-231 cells were transfected using FuGENE 6 according to the manufacturer’s instruction.

### Real-Time Quantitative RT-PCR

Total RNA was isolated from cell lines using TRIzol reagent (Invitrogen, Inc.) under the manufacturer’s protocols. Then the RNA was reversed transcribed to cDNA using SuperScript Reverse Transcriptase (Takara, Shiga, Japan). Reactions were performed using the SYBR Green PCR Kit (Takara, Japan). GAPDH was used as an internal control. The mRNA expression folds were analyzed by 2^-ΔΔ^ *^C^* ^t^. The primer sequences were as follows: Ezrin-forward: 5′-CGCTCTAAGGTTCTGCTCT-3′, Ezrin-reverse: 5′-TCCTGGGCAGACACCTTCTTA-3′; GAPDH-forward: 5′-CTGGGCTACACTGAGCACC-3′, GAPDH-reverse: 5′-AAGTGGTCGTTGAGGGCAATG-3′. Each experiment was conducted at least three times.

### Phalloidin Staining of F-Actin

On the first day, stable cell lines were transferred to 24 wells. 24 hours later, they were fixed with paraformaldehyde for 10 min and permeabilized with 1 ml 0.2% of Triton X-100 for 10 min. After three times of PBS washing, samples were blocked with 1% of bovine serum albumin (BSA; Sigma-Aldrich, St. Louis, MO, USA) and incubated with 100 μl fluorescent phalloidin (Phalloidin-iFluor 488 Reagent, Abcam, ab176753) for 1 h, stained with DAPI for 10 min. Both of them were put in the dark environment at room temperature. After 3 times of PBS washing, cells were observed under a confocal microscope (Olympus, Center Valley, PA, USA).

### Enzyme-Linked Immunosorbent Assay (ELISA)

The ELISA technique kit (Cusabio Biotech, Wuhan, China) was used to evaluate Ezrin expression in different AJAP1 expression groups according to the manufacturer’s guidelines. Eventually, both of them were assessed using a spectrophotometer (Thermo Scientific, Waltham, MA, USA) at 450 nm.

### Bioinformatics Analysis

StarBase V3.0 (https://www.ncbi.nlm.nih.gov/geo/) online databases were used to validate the potential relationship between AJAP1 and Ezrin in breast cancer. StarBase V3.0 is an open-source platform for studying the miRNA–ncRNA, miRNA–mRNA, ncRNA–RNA, RNA–RNA, RBP–ncRNA, and RBP–mRNA interactions from CLIP-seq, degradome-seq, and RNA–RNA interactome data. Besides, it also allows researchers to perform Pan-Cancer analysis on RNA–RNA and RBP–RNA interactions, as well as the survival and differential expression analysis of miRNAs, lncRNAs, pseudogenes, and mRNAs.

Then we also downloaded breast cancer datasets from the Cancer Genome Atlas (TCGA) project and used “R” software to analyze the potential relationship between AJAP1 and Ezrin expression.

### Statistical Analysis

Statistical analyses were conducted using SPSS24.0 software. Clinicopathological parameters with the expression of two proteins were evaluated by the chi-square test and spearman test. Kaplan–Meier curves of DFS and OS were constructed. All data were shown as mean ± S.D. *p* < 0.05 was considered statistically significant.

## Results

### Expression of Ezrin and AJAP1 in Breast Cancer

**Figure 1:**
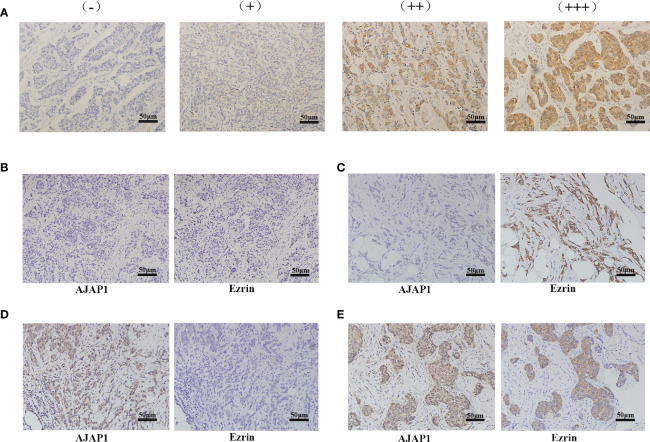
Different expression of Ezrin and AJAP1 in breast cancer. **(A)** Different staining intensity of Ezrin in breast cancer (×100). **(B–E)** Series slides of Ezrin and AJAP1 expression in breast cancer tissue slides: **(B)** AJAP1 negative/Ezrin-negative (×100), **(C)** AJAP1-negative/Ezrin-positive (×100), **(D)** AJAP1-positive/Ezrin-negative (×100), **(E)** AJAP1-positive/Ezrin-positive (×100).

AJAP1 and Ezrin expressions were detected in 377 cases of breast cancer using immunohistochemistry (IHC) technology. Positive staining of Ezrin was mainly observed in the cytoplasm in the breast cancer tissue slides. Different staining intensities of Ezrin are demonstrated in **Figure 1A**. As our previous study reported (13), AJAP1 was mainly located in the cytoplasm, with little membrane staining. There are four different expression patterns between AJAP1 and Ezrin (**Figures 1B**–**E**). Thus, AJAP1-positive staining was observed in 213 (56.5%) cases of 377 breast cancer samples and Ezrin-positive staining occurred in 165 (43.77%) cases of 377 breast cancer tissue slides.

### Correlation Between Ezrin and AJAP1 Expression With Clinicopathological Parameters

Next, the association between AJAP1 expression and Ezrin expression and the clinicopathological characteristics of breast cancer patients are shown in **Table 1**. It was obvious that AJAP1 expression was closely associated with histological grade (*p* < 0.0001) and lymph node (*p* < 0.0001). Meanwhile, Ezrin expression was more closely related with histological grade (*p* = 0.004) and lymph node (*p* < 0.0001). However, other clinicopathological parameters did not show any significant association with AJAP1 expression or Ezrin expression.

**Table 1:** AJAP1 and Ezrin expression with clinicopathological parameters in breast cancer patients.

Factor	AJAP1				*p*-value	Ezrin				*p*-value
	Positive		Negative			Positive		Negative		
	N	%	N	%		N	%	N	%	
All	213	56.50	164	43.50		165	43.77	212	56.23	
**Age**										
≤50	104	48.8	81	49.4	0.914	86	52.1	99	46.7	0.296
>50	109	51.2	83	50.6		79	47.9	113	53.3	
**Menopausal status**										
Premenopausal	115	54.0	87	53.0	0.856	97	58.8	105	49.5	0.074
Postmenopausal	98	46.0	77	47.0		68	41.2	107	50.5	
**Tumor size**										
T1	93	43.7	63	38.4	0.250	62	37.6	94	44.3	0.372
T2	102	47.9	79	48.2		83	50.3	98	46.2	
T3	18	8.5	22	13.4		20	12.1	20	9.4	
**Histological grade**										
1	53	24.9	16	9.8	<0.0001^*^	23	13.9	46	21.7	0.004^*^
2	117	54.9	83	50.6		81	49.1	119	56.1	
3	43	20.2	65	39.6		61	37.0	47	22.2	
**Lymph node**										
0	166	77.9	23	14.0	<0.0001^*^	56	33.9	133	62.7	<0.0001^*^
1–3	34	16.0	65	39.6		37	22.4	62	29.2	
4–9	9	4.2	50	30.5		45	27.3	14	6.6	
≥10	4	1.9	26	15.9		27	16.4	3	1.4	
**ER**										
Negative	82	38.5	66	40.2	0.731	67	40.6	81	38.2	0.636
Positive	131	61.5	98	59.8		98	59.4	131	61.8	
**PR**										
Negative	112	52.6	76	46.3	0.230	77	46.7	111	52.4	0.273
Positive	101	47.4	88	53.7		88	53.3	101	47.6	
**Her-2**										
Negative	161	75.6	122	74.4	0.790	119	72.1	164	77.4	0.244
Positive	52	24.4	42	25.6		46	27.9	48	22.6	
**Ki67**										
<20	48	22.5	28	17.1	0.190	33	20.0	43	20.3	0.946
≥20	165	77.5	136	82.9		132	80.0	169	79.7	
**P53**										
Negative	103	48.4	86	52.4	0.432	82	49.7	107	50.5	0.881
Positive	110	51.6	78	47.6		83	50.3	105	49.5	

*Difference was statistically significant.

### Different Expressional Patterns of AJAP1 and Ezrin With Clinicopathological Parameters

**Figure 2:**
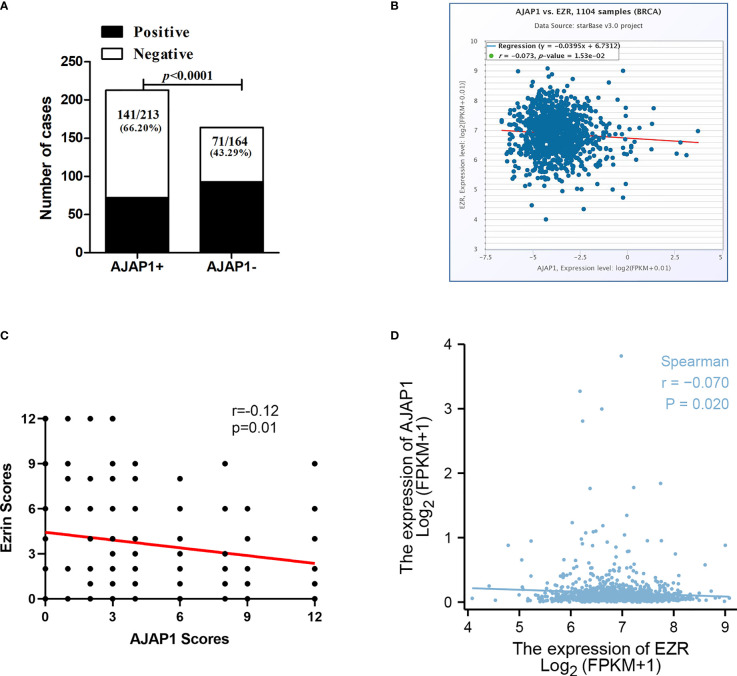
AJAP1 expression is negatively correlated with Ezrin expression. **(A)** Number of cases of different Ezrin expression in AJAP1+ and AJAP1- samples. **(B)** Scatter plot analysis for the correlation between AJAP1 and Ezrin based on starBase v3.0. **(C)** Spearman test about the relationship between AJAP1 and Ezrin in 377 breast cancer cases. **(D)** Spearman analysis for the correlation between AJAP1 and Ezrin based on TCGA datasets.

**Table 2** shows that the different expressional patterns’ results were inconsistent with the former results and they were also associated with lymph node (*p* < 0.0001) and histological grade (*p* < 0.001). Next, **Figure 2A** also shows that AJAP1 expression was also related with Ezrin expression. What is more, starBase v3.0 was utilized to reveal that AJAP1 expression was negatively related with Ezrin expression (**Figure 2B**). We also used Spearman test to analyze the relationship between AJAP1 and Ezrin. **Figure 2C** shows that AJAP1 was inversely related with Ezrin expression in 377 cases of breast cancer. The data of the TCGA dataset also identified this (**Figure 2D**).

**Table 2:** AJAP1/Ezrin expression and clinicopathological parameter in patients with breast cancer patients.

Factor	AJAP1+Ezrin+		AJAP1+/Ezrin-		AJAP1-/Ezrin+		AJAP1-/Ezrin-		*p* value
	N	%	N	%	N	%	N	%	
**All**	72	19.10	141	37.40	93	24.67	71	18.83	
**Age**									
≤50	38	52.8	66	46.8	48	51.6	33	46.5	0.773
>50	34	47.2	75	53.2	45	48.4	38	53.5	
**Menopausal status**									
Premenopausal	43	59.7	72	51.1	54	58.1	33	46.5	0.303
Postmenopausal	29	40.3	69	48.9	39	41.9	38	53.5	
**Tumor size**									
T1	29	40.3	64	45.4	33	35.5	30	42.3	0.663
T2	36	50.0	66	46.8	47	50.5	32	45.1	
T3	7	9.7	11	7.8	13	14.0	9	12.7	
**Histological grade**									
1	17	23.6	36	25.5	6	6.5	10	14.1	<0.0001^*^
2	37	51.4	80	56.7	44	47.3	39	54.9	
3	18	25.0	25	17.7	43	46.2	22	31.0	
**Lymph node**									
0	54	75.0	112	79.4	2	2.2	21	29.6	<0.0001^*^
1–3	10	13.9	24	17.0	27	29.0	38	53.5	
4–9	5	6.9	4	2.8	40	43.0	10	14.1	
≥10	3	4.2	1	0.7	24	25.8	2	2.8	
**ER**									
Negative	25	34.7	57	40.4	42	45.2	24	33.8	0.400
Positive	47	65.3	84	59.6	51	54.8	47	66.2	
**PR**									
Negative	33	45.8	79	56.0	44	47.3	32	45.1	0.320
Positive	39	54.2	62	44.0	49	52.7	39	54.9	
**Her-2**									
Negative	51	70.8	110	78.0	68	73.1	54	76.1	0.666
Positive	21	29.2	31	22.0	25	26.9	17	23.9	
**Ki67**									
<20	18	25.0	30	21.3	83.9	16.1	13	18.3	0.523
≥20	54	75.0	111	78.7	78	83.9	58	81.7	
**P53**									
Negative	35	48.6	68	48.2	47	50.5	39	54.9	0.818
Positive	37	51.4	73	51.8	46	49.5	32	45.1	

*Difference was statistically significant.

### Survival Analysis

**Figure 3:**
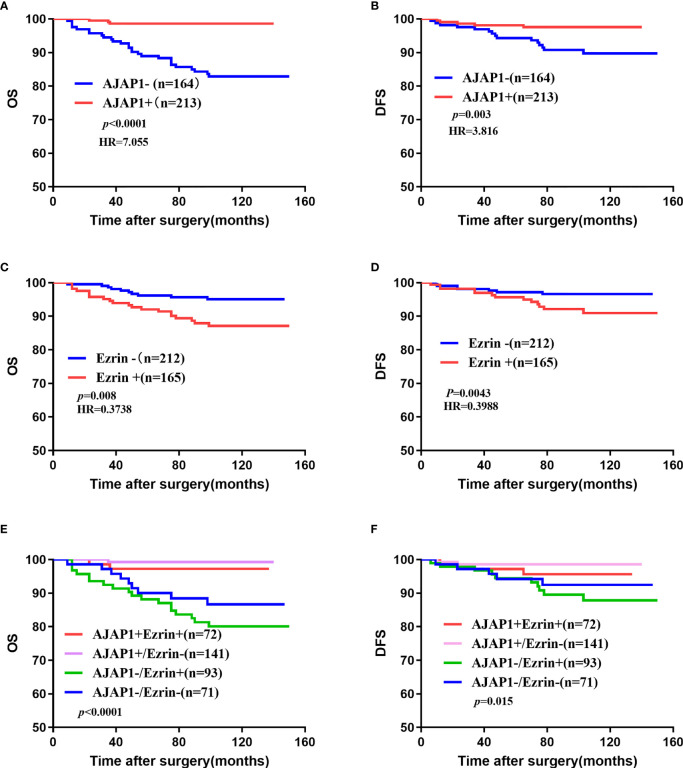
Overall survival and disease-free survival of 377 cases of breast cancer. **(A, B)** OS **(A)** and DFS **(B)** of different AJAP1 expression. **(C, D)** OS **(A)** and DFS **(B)** of different Ezrin expression. **(E, F)** OS **(E)** and DFS **(F)** of different combination between AJAP1 and Ezrin expression.

Kaplan–Meier curves demonstrated that a high expression of AJAP1 showed a good prognosis and short disease progression (**Figure 3A**, OS: *p* < 0.0001; **Figure 3B**, DFS: *p* = 0.003). The OS and DFS curves showed that Ezrin expression was associated with shorter OS (*p* = 0.008, **Figure 3C**) and DFS (*p* = 0.0043, **Figure 3D**). The results of expression patterns with OS and DFS demonstrated that tumors with AJAP1-Ezrin+ expression exhibited the worst OS (*p* < 0.0001, **Figure 3E**) and shortest DFS (*p* = 0.015, **Figure 3F**) among four groups.

What is more, univariate analysis (**Table 3**) demonstrated that AJAP1-/Ezrin+ was a significant risk factor for unfavorable prognosis of OS (*p* = 0.005) and DFS (*p* = 0.044). Histological grade and lymph node metastasis also showed poor OS (*p* < 0.0001 and *p* < 0.0001, respectively) and short DFS (*p* = 0.031 and *p* = 0.003, respectively) among the four groups. Other factors did not have significant difference.

**Table 3:** Univariate analysis of OS and DFS in breast cancer patients.

Factors	OS	*p*	DFS	*p*
	HR (95% CI)		HR (95% CI)	
**AJAP1/Ezrin**				
AJAP1+/Ezrin+	1		1	
AJAP1+/Ezrin-	0.219(0.047,1.015)	0.052	0.593(0.142,2.481)	0.474
AJAP1-/Ezrin+	0.052(0.007-0.414)	0.005*	0.186(0.036,0.959)	0.044*
AJAP1-/Ezrin-	1.533(0.689-3.414)	0.295	1.509(0.516,4.417)	0.452
**Age**				
≤50	1		1	
>50	1.286(0.624,2.647)	0.495	0.983(0.409,2.361)	0.969
**Menopausal status**				
Premenopausal	1		1	
Postmenopausal	1.160(0.567,2.372)	0.685	0.627(0.250,1.571)	0.319
**Tumor size**				
T1	0.540(0.205-1.420)	0.211	0.670(0.178,2.525)	0.670
T2	0.401(0.148,1.085)	0.072	0.672(0.182,2.484)	0.672
T3	1		1	
**Histological grade**				
1–2	1		1	
3	5.256(2.460,11.230)	<0.0001*	0.382(0.159,0.918)	0.031*
**Lymph node**				
Yes	10.255(4.563,23.046)	<0.0001*	3.723(1.548,8.954)	0.003*
No	1		1	
**ER**				
Positive	1		1	
Negative	0.873(0.610,1.250)	0.460	1.255(0.778,2.024)	0.353
**PR**				
Positive	1		1	
Negative	1.014(0.496,2.075)	0.969	2.049(0.925,6.269)	0.072
**Her-2**				
Positive	1		1	
Negative	1.711(0.824,3.554)	0.150	0.687(0.426,1.108)	0.123
**Ki67**				
<20	1		1	
≥20	2.714(1.002,7.353)	0.05	1.520(0.732,3.156)	0.261
**P53**				
Positive	1		1	
Negative	0.750(0.518,1.088)	0.129	0.802(0.512,1.254)	0.333

OS, overall survival; DFS, disease-free survival.

*Difference was statistically significant.

Multivariate analysis (**Table 4**) showed that AJAP1-Ezrin+, histological grade, and lymph node metastasis were risk factors for OS (*p* = 0.021, *p* = 0.005, and *p* < 0.0001). Additionally, none of the factors showed a significant difference.

**Table 4:** Multivariate analysis of OS and DFS in breast cancer patients.

Factors	OS	*p*	DFS	*p*
	HR (95% CI)		HR (95% CI)	
**AJAP1/Ezrin**				
AJAP1+/Ezrin+	1			
AJAP1+/Ezrin-	0.250 (0.054,1.158)	0.076	0.612(0.146,2.562)	0.510
AJAP1-/Ezrin+	0.086(0.011,0.690)	0.021*	0.227(0.043,1.188)	0.079
AJAP1-/Ezrin-	0.851(0.375,1.932)	0.699	1.089(0.359,3.309)	0.880
**Histological grade**				
1–2	0.333(0.154,0.720)	0.005*	0.559(0.227,1.378)	0.206
3	1		1	
**Lymph node**				
Yes	1		1	
No	0.191(0.082,0.446)	<0.0001*	0.445(0.173,1.144)	0.093

OS, overall survival; DFS, disease-free survival.

*Difference was statistically significant.

Taken together, AJAP1-Ezrin+ was a potential risk factor for predicting breast cancer patients with poor prognosis.

### Evaluation of Diagnostic Efficiency

**Figure 4:**
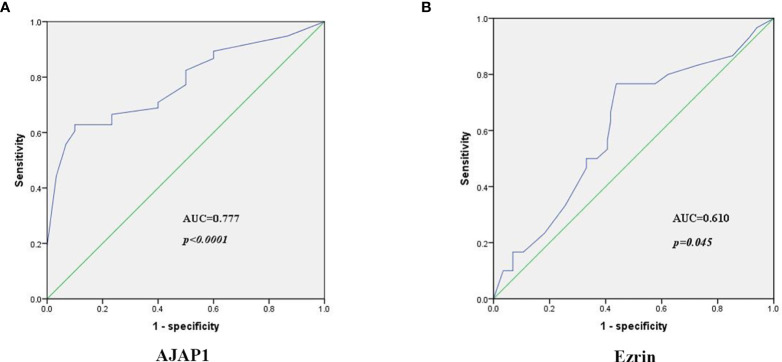
The diagnosis power of AJAP1 **(A)** and Ezrin **(B)** expression in breast cancer patients.

Next, ROC curves and the area under the curve (AUC) were used to assess the accuracy of AJAP1 and Ezrin expressions as biomarkers for breast cancer diagnosis. Results demonstrated that AJAP1 AUC was 0.777 (95% confidence interval was 0.711–0.844, *p* < 0.0001) in different breast cancer patients and the optimal cutoff value was 0.528 (**Figure 4A**). Meanwhile, Ezrin’s AUC was 0.610 (95% confidence interval was 0.507–0.714, *p* = 0.045) with the optical cutoff value of 0.329 (**Figure 4B**).

Next, the sensitivity and specificity of AJAP1 expression and Ezrin expression were calculated using AJAP1 expression 0.528 and Ezrin expression 0.329 as the cutoff. AJAP1 expression specificity and sensitivity were 0.561 and 0.767, respectively. As for Ezrin expression, the corresponding values were 0.9 and 0.628, respectively.

### AJAP1 Affects the Cytoskeleton of Breast Cancer Cell by Mediating Ezrin Expression

**Figure 5:**
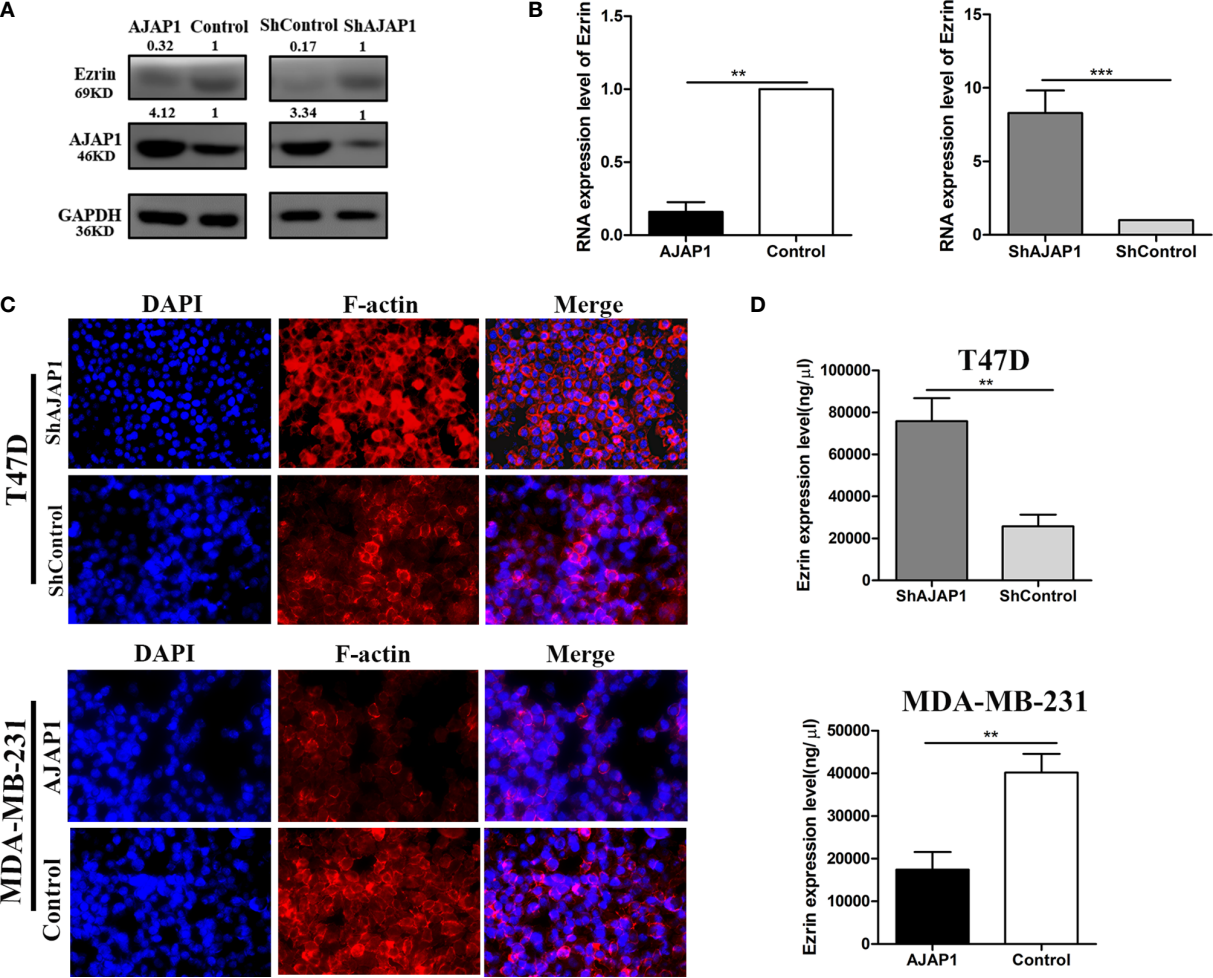
AJAP1 inhibits cytoskeleton formation by reducing Ezrin expression. **(A)** Western blot results of Ezrin expression in AJAP1-overexpressed MDA-MB-231 cells and AJAP1-silenced T47D cells. **(B)** qRT-PCR results of Ezrin expression in AJAP1 overexpressed MDA-MB-231 cells and AJAP1-silenced T47D cells. **(C)** Fluorescent staining of F-actin by phalloidin in AJAP1 depletion T47D cells and AJAP1 overexpressed MDA-MB-231 cells. **(D)** The Ezrin level changes in different expression of AJAP1 stable cell lines by ELISA. Data were shown as mean ± SD. Each experiment was conducted at least three times **p < 0.01, ***p < 0.001.

The above results showed that AJAP1 expression was negatively related with Ezrin expression in breast cancer tissue slides. Besides, both AJAP1 and Ezrin were important molecules that maintained the cell structure and actin cytoskeleton. Next, the effect of changing the AJAP1 expression on the cytoskeleton of breast cancer cells was explored. Firstly, we conducted T47D cells with a knocked-down AJAP1 expression and MDA-MB-231 with an overexpressed AJAP1 expression. Results of the Western blot showed that AJAP1 depletion promoted Ezrin expression in T47D and upregulated AJAP1 exhibited the opposite results in MDA-MB-231 cells (**Figure 5A**). Then, qRT-PCR was also conducted to observe the RNA levels in cells with different expressions of AJAP1 (**Figure 5B**). It seemed that overexpressed AJAP1 can reduce the Ezrin RNA level and downregulation of AJAP1 increased the Ezrin RNA level, while when we overexpressed or knocked down Ezrin expression, it had no effect on AJAP1 (**Supplementary Figure S1**). Collectively, AJAP1 negatively mediated Ezrin expression in breast cancer cell lines. After that, the effect of changing the AJAP1 expression on the cytoskeleton of breast cancer cells was explored. The results of fluorescent staining of F-actin through phalloidin showed that AJAP1 depletion in T47D cells increased the amount of F-actin expression in the cytoskeleton filaments, which is demonstrated by a significant increase in fluorescent intensity compared with ShControl groups (**Figure 5C**). Consistently, ELISA assay showed a significant increase in Ezrin expression level after silencing AJAP1 expression in T47D cells (**Figure 5D**, top panel). Apart from this, overexpressed AJAP1 expression in MDA-MB-231 cells revealed contrasting results (**Figures 5C, D**). These experiments together suggested that AJAP1 suppressed actin expression by promoting Ezrin expression.

## Discussion

Tumor metastasis and invasion are a series of complex, multistep progression that depends on the dynamic motion of cell to cell and cell to extracellular matrix. Besides, the key factor is cytoskeleton-related proteins. Ezrin belongs to the ERM (ezrin–radixin–moesin) family, located on 6q25.2-q26 (17). Most studies also demonstrated that it was a tumor metastasis mediator (28). In breast cancer, many reports revealed Ezrin’s different functions. Li et al. (29) revealed that upregulated Ezrin expression was positively related with lymph node involvement and proved that Ezrin could be a biomarker for predicting lymphatic metastasis of invasive ductal carcinoma. Consistent with their results, we showed that Ezrin expression was related with lymph node involvement and histological grade as well. As for breast cancer prognosis, high expression of Ezrin predicted poor OS and high DFS. Besides, David et al. (25) analyzed different locations of Ezrin and summarized that loss of Ezrin apical polarization was related with adverse tumor characteristics of breast cancer cells. Complete membrane staining of Ezrin was linked to high-grade, strong Her-2 and p-AKT expression. In this study, any significant relation between Ezrin expression and Her-2 expression had not been observed due to the limited number of samples. Moreover, silencing of Ezrin reduced the ability of breast cancer cell motion and invasion. Besides, many reports showed that estrogen E2, CD44, etc., mediated Ezrin to promote the malignant potential of breast cancer (30, 31). What is more, Ezrin is also related with breast cancer multidrug resistance (32, 33). It was found in the study that microparticles from breast cancer had tissue selectivity, that is to say, they only transferred resistance proteins to malignant breast cells. ERM protein family and cytoskeletal dynamic proteins may be one of the mechanisms for the multidrug resistance of microparticles from breast cancer (34).

Cell adhesion molecules are glycoproteins which link to the metastasis of tumor cells and that have been extensively studied in recent years. They are mainly distributed on the surface of the cell membrane, and their main function is to regulate the adhesion ability between cells and matrix. AJAP1 is a novel protein of adherens junction and has also been explored in 377 samples of breast cancer tissues. Our study found that the AJAP1-positive rate in 377 samples is 56.5% (213/377) and a low expression of AJAP1 also positively associated with histological grade and lymph node. AJAP1 expression was negatively associated with Ezrin expression including the prognosis function for breast cancer patients. A number of studies on AJAP1 vital function in a variety of types of cancer have attracted people’s attention (5–12, 35–39). Moreover, our report provided the first document to explore the relationship between AJAP1 and Ezrin expression in breast cancer tissue slides and analyzed their expression with clinicopathological parameters.

During the past decades, many tools and markers were found to reflect the prognosis of breast cancer and created great advantage on daily clinical work (40–43). In our research, we found that AJAP1 expression was negatively linked with Ezrin expression and their combination can predict the prognosis of breast cancer. However, the ROC curve demonstrated that AJAP1 showed more accuracy to evaluate the OS status than Ezrin expression. Thus, the results for the combination of AJAP1 and Ezrin expression showed that AJAP1^negative^ Ezrin^positive^ tended to have a low OS. Meanwhile, univariate and multivariate analyses demonstrated that AJAP1^negative^Ezrin^positive^ was a potential risk factor for breast cancer patients’ OS.

The proliferation of tumor cells depends on cytoskeletal recombination, formation of filamentous actin (actin) stress fibers, and increased cytoskeletal protein content. All of them may become the key to influencing the occurrence of cancer invasion and metastasis. Previous studies showed that AJAP1 controlled cell cytoskeleton to inhibit the tumor progression of glioma. Thus, we next examined the effect of AJAP1 act on the cytoskeleton. Here, in our study, we found that AJAP1 depletion can reduce the expression of F-actin. Besides, we also detected the level of Ezrin in AJAP1-silencing cells by ELISA and found that downregulation of AJAP1 can reduce the Ezrin expression. Therefore, we presumed that AJAP1 may have prevented tumor malignant behavior by inhibiting Ezrin expression. However, the concrete mechanism needed to be explored in the future days.

To sum up, our research revealed that AJAP1 was low expressed in breast cancer and elucidated its potential pivotal biological role as well. Besides, we also demonstrated a new relationship between AJAP1 and Ezrin in mediating the cytoskeleton of breast cancer cells. However, further studies were needed to analyze the concrete pathway between AJAP1-mediated Ezrin activity in prohibiting breast cancer progression and related clinical therapeutic strategies.

## Data Availability Statement

The original contributions presented in the study are included in the article/**Supplementary Material**. Further inquiries can be directed to the corresponding authors.

## Ethics Statement

This study was approved by the Tianjin Medical University Cancer Institute and Hospital, China, and has been performed in accordance with the ethical standards laid down in the 1975 Helsinki Declaration, revised in 2013. The patients/participants provided their written informed consent to participate in this study.

## Author Contributions

XHH,YYP and YN contributed equally to this work and shared last authorship. All authors contributed to the article and approved the submitted version.

## Funding

This study was funded by the Key Research and Development Projects from Science and Technology Department of Anhui Province (1704a0802148 and 1804h08020259) and the Hefei Municipal Independent Innovation Policy “Borrowing and Transferring” Project (J2018Y01).

## Conflict of Interest

The authors declare that the research was conducted in the absence of any commercial or financial relationships that could be construed as a potential conflict of interest.

## Publisher’s Note

All claims expressed in this article are solely those of the authors and do not necessarily represent those of their affiliated organizations, or those of the publisher, the editors and the reviewers. Any product that may be evaluated in this article, or claim that may be made by its manufacturer, is not guaranteed or endorsed by the publisher.

## Supplementary Material

Supplementary Figure 1 qRT-PCR results of AJAP1 expression in Ezrin overexpressed MDA-MB-231 cells and Ezrin-silenced T47D cells. N.S., no significance.

## Abbreviations

AJAP1, adherens junction-associated protein 1; IHC, immunohistochemistry; qRT-PCR, quantitative reverse-transcription polymerase chain reaction; ELISA, enzyme-linked immunosorbent assay; OS, overall survival; DFS, disease-free survival.

## References

[1] Ginsburg O Bray F Coleman MP Vanderpuye V Eniu A Kotha SR. The Global Burden of Women’s Cancers: A Grand Challenge in Global Health. Lancet (2017) 389(10071):847–60. doi: 10.1016/S0140-6736(16)31392-7 PMC6191029 27814965

[2] Meng W Takeichi M. Adherens Junction: Molecular Architecture and Regulation. Cold Spring Harbor Perspect Biol (2009) 1(6):a002899. doi: 10.1101/cshperspect.a002899 PMC2882120 20457565

[3] Zeng L Fee BE Rivas MV Lin J Adamson DC. Adherens Junctional Associated Protein-1: A Novel 1p36 Tumor Suppressor Candidate in Gliomas (Review). Int J Oncol (2014) 45(1):13–7. doi: 10.3892/ijo.2014.2425 24807315

[4] Bharti S Handrow-Metzmacher H Zickenheiner S Zeitvogel A Baumann R Starzinski-Powitz A. Novel Membrane Protein Shrew-1 Targets to Cadherin-Mediated Junctions in Polarized Epithelial Cells. Mol Biol Cell (2004) 15(1):397–406. doi: 10.1091/mbc.e03-05-0281 14595118 PMC307556

[5] Yang C Li YS Wang QX Huang K Wei JW Wang YF. EGFR/EGFRvIII Remodels the Cytoskeleton *via* Epigenetic Silencing of AJAP1 in Glioma Cells. Cancer Lett (2017) 403:119–27. doi: 10.1016/j.canlet.2017.06.007 28634045

[6] Di C Mladkova N Lin J Fee B Rivas M Chunsheng K. AJAP1 Expression Modulates Glioma Cell Motility and Correlates With Tumor Growth and Survival. Int J Oncol (2018) 52(1):47–54. doi: 10.3892/ijo.2017.4184 29115565 PMC5743336

[7] Han L Zhang KL Zhang JX Zeng L Di CH Fee BE. AJAP1 is Dysregulated at an Early Stage of Gliomagenesis and Suppresses Invasion Through Cytoskeleton Reorganization. CNS Neurosci Ther (2014) 20(5):429–37. doi: 10.1111/cns.12232 PMC4511491 24483339

[8] Han J Xie C Pei T Wang J Lan Y Huang K. Deregulated AJAP1/beta-Catenin/ZEB1 Signaling Promotes Hepatocellular Carcinoma Carcinogenesis and Metastasis. Cell Death Dis (2017) 8(4):e2736. doi: 10.1038/cddis.2017.126 28383563 PMC5477574

[9] Qu W Wen X Su K Gou W. MiR-552 Promotes the Proliferation, Migration and EMT of Hepatocellular Carcinoma Cells by Inhibiting AJAP1 Expression. J Cell Mol Med (2019) 23(2):1541–52. doi: 10.1111/jcmm.14062 PMC6349347 30597727

[10] Tanaka H Kanda M Koike M Iwata N Shimizu D Ezaka K. Adherens Junctions Associated Protein 1 Serves as a Predictor of Recurrence of Squamous Cell Carcinoma of the Esophagus. Int J Oncol (2015) 47(5):1811–8. doi: 10.3892/ijo.2015.3167 26397940

[11] McDonald JM Dunlap S Cogdell D Dunmire V Wei Q Starzinski-Powitz A. The SHREW1 Gene, Frequently Deleted in Oligodendrogliomas, Functions to Inhibit Cell Adhesion and Migration. Cancer Biol Ther (2006) 5(3):300–4. doi: 10.4161/cbt.5.3.2391 16410724

[12] Hotte K Smyrek I Starzinski-Powitz A Stelzer EHK. Endogenous AJAP1 Associates With the Cytoskeleton and Attenuates Angiogenesis in Endothelial Cells. Biol Open (2017) 6(6):723–31. doi: 10.1242/bio.022335 PMC5483013 28483980

[13] Xu C Liu F Xiang G Cao L Wang S Liu J. Beta-Catenin Nuclear Localization Positively Feeds Back on EGF/EGFR-Attenuated AJAP1 Expression in Breast Cancer. J Exp Clin Cancer Res: CR (2019) 38(1):238. doi: 10.1186/s13046-019-1252-6 31171012 PMC6554977

[14] Ogihara T Mizoi K Kamioka H Yano K. Physiological Roles of ERM Proteins and Transcriptional Regulators in Supporting Membrane Expression of Efflux Transporters as Factors of Drug Resistance in Cancer. Cancers (2020) 12(11):3352. doi: 10.3390/cancers12113352 PMC7696277 33198344

[15] Clucas J Valderrama F. ERM Proteins in Cancer Progression. J Cell Sci (2014) 127(Pt 2):267–75. doi: 10.1242/jcs.133108 24421310

[16] Hashimoto K Hayashi R Mukaigawa T Yamazaki M Fujii S. Concomitant Expression of Ezrin and HER2 Predicts Distant Metastasis and Poor Prognosis of Patients With Salivary Gland Carcinomas. Hum Pathol (2017) 63:110–9. doi: 10.1016/j.humpath.2017.02.017 28300573

[17] Derouiche A Geiger KD. Perspectives for Ezrin and Radixin in Astrocytes: Kinases, Functions and Pathology. Int J Mol Sci (2019) 20(15):3776. doi: 10.3390/ijms20153776 PMC6695708 31382374

[18] Song Y Ma X Zhang M Wang M Wang G Ye Y. Ezrin Mediates Invasion and Metastasis in Tumorigenesis: A Review. Front Cell Dev Biol (2020) 8:588801. doi: 10.3389/fcell.2020.588801 33240887 PMC7683424

[19] Bretscher A Edwards K Fehon RG. ERM Proteins and Merlin: Integrators at the Cell Cortex. Nat Rev Mol Cell Biol (2002) 3(8):586–99. doi: 10.1038/nrm882 12154370

[20] Ponuwei GA. A Glimpse of the ERM Proteins. J Biomed Sci (2016) 23:35. doi: 10.1186/s12929-016-0246-3 26983550 PMC4794931

[21] Piao J Liu S Xu Y Wang C Lin Z Qin Y. Ezrin Protein Overexpression Predicts the Poor Prognosis of Pancreatic Ductal Adenocarcinomas. Exp Mol Pathol (2015) 98(1):1–6. doi: 10.1016/j.yexmp.2014.11.003 25445504

[22] Horwitz V Davidson B Stern D Trope CG Tavor Re’em T Reich R. Ezrin Is Associated With Disease Progression in Ovarian Carcinoma. PloS One (2016) 11(9):e0162502. doi: 10.1371/journal.pone.0162502 27622508 PMC5021292

[23] Zhang XQ Chen GP Wu T Yan JP Zhou JY. Expression and Clinical Significance of Ezrin in non–Small-Cell Lung Cancer. Clin Lung Cancer (2012) 13(3):196–204. doi: 10.1016/j.cllc.2011.04.002 22137559

[24] Wang L Lin GN Jiang XL Lu Y. Expression of Ezrin Correlates With Poor Prognosis of Nasopharyngeal Carcinoma. Tumour Biol: J Int Soc Oncodevelopmental Biol Med (2011) 32(4):707–12. doi: 10.1007/s13277-011-0171-8 21625943

[25] Sarrio D Rodriguez-Pinilla SM Dotor A Calero F Hardisson D Palacios J. Abnormal Ezrin Localization is Associated With Clinicopathological Features in Invasive Breast Carcinomas. Breast Cancer Res Treat (2006) 98(1):71–9. doi: 10.1007/s10549-005-9133-4 16538541

[26] Li L Wang YY Zhao ZS Ma J. Ezrin is Associated With Gastric Cancer Progression and Prognosis. Pathol Oncol Res: POR (2011) 17(4):909–15. doi: 10.1007/s12253-011-9402-y 21717114

[27] Li N Kong J Lin Z Yang Y Jin T Xu M. Ezrin Promotes Breast Cancer Progression by Modulating AKT Signals. Br J Cancer (2019) 120(7):703–13. doi: 10.1038/s41416-019-0383-z PMC6461860 30804430

[28] Bruce B Khanna G Ren L Landberg G Jirstrom K Powell C. Expression of the Cytoskeleton Linker Protein Ezrin in Human Cancers. Clin Exp Metastasis (2007) 24(2):69–78. doi: 10.1007/s10585-006-9050-x 17370041

[29] Ma L Zhang XH Xing LX Li YH Wang XL Wang YJ. Relationship of Ezrin Protein Expression to the Carcinogenesis and Prognosis of Infitrating Breast Ductal Carcinoma. Zhonghua Zhong Liu Za Zhi Chinese J Oncol (2008) 30(4):279–83. 18788632

[30] Pokharel D Padula MP Lu JF Jaiswal R Djordjevic SP Bebawy M. The Role of CD44 and ERM Proteins in Expression and Functionality of P-Glycoprotein in Breast Cancer Cells. Molecules (2016) 21(3):290. doi: 10.3390/molecules21030290 26938523 PMC6273996

[31] Nam K Oh S Lee KM Yoo SA Shin I. CD44 Regulates Cell Proliferation, Migration, and Invasion *via* Modulation of C-Src Transcription in Human Breast Cancer Cells. Cell Signalling (2015) 27(9):1882–94. doi: 10.1016/j.cellsig.2015.05.002 25979842

[32] Ma L Liu YP Geng CZ Xing LX Zhang XH. Low-Dose Epirubicin Inhibits Ezrin-Mediated Metastatic Behavior of Breast Cancer Cells. Tumori (2011) 97(3):400–5. doi: 10.1177/030089161109700324 21789023

[33] Li J Tu Y Wen J Yao F Wei W Sun S. Role for Ezrin in Breast Cancer Cell Chemotaxis to CCL5. Oncol Rep (2010) 24(4):965–71. doi: 10.3892/or.2010.965 20811677

[34] Jaiswal R Luk F Dalla PV Grau GE Bebawy M. Breast Cancer-Derived Microparticles Display Tissue Selectivity in the Transfer of Resistance Proteins to Cells. PloS One (2013) 8(4):e61515. doi: 10.1371/journal.pone.0061515 23593486 PMC3625154

[35] Zeng L Kang C Di C Fee BE Rivas M Lin J. The Adherens Junction-Associated Protein 1 is a Negative Transcriptional Regulator of MAGEA2, Which Potentiates Temozolomide-Induced Apoptosis in GBM. Int J Oncol (2014) 44(4):1243–51. doi: 10.3892/ijo.2014.2277 24481586

[36] Schreiner A Ruonala M Jakob V Suthaus J Boles E Wouters F. Junction Protein Shrew-1 Influences Cell Invasion and Interacts With Invasion-Promoting Protein CD147. Mol Biol Cell (2007) 18(4):1272–81. doi: 10.1091/mbc.e06-07-0637 PMC1838978 17267690

[37] Resch E Quaiser S Quaiser T Schneider G Starzinski-Powitz A Schreiner A. Synergism of Shrew-1’s Signal Peptide and Transmembrane Segment Required for Plasma Membrane Localization. Traffic (2008) 9(8):1344–53. doi: 10.1111/j.1600-0854.2008.00765.x 18485053

[38] Gross JC Schreiner A Engels K Starzinski-Powitz A. E-Cadherin Surface Levels in Epithelial Growth Factor-Stimulated Cells Depend on Adherens Junction Protein Shrew-1. Mol Biol Cell (2009) 20(15):3598–607. doi: 10.1091/mbc.e08-12-1240 PMC2719577 19515834

[39] Cogdell D Chung W Liu Y McDonald JM Aldape K Issa JP. Tumor-Associated Methylation of the Putative Tumor Suppressor AJAP1 Gene and Association Between Decreased AJAP1 Expression and Shorter Survival in Patients With Glioma. Chin J Cancer (2011) 30(4):247–53. doi: 10.5732/cjc.011.10025 PMC4013351 21439246

[40] Zhou P Zhang WW Bao Y Wang J Lian CL He ZY. Chemotherapy and 21-Gene Recurrence Score Testing for Older Breast Cancer Patients: A Competing-Risks Analysis. Breast (2020) 54:319–27. doi: 10.1016/j.breast.2020.11.018 PMC7718160 33278648

[41] Yousefi H Vatanmakanian M Mahdiannasser M Mashouri L Alahari NV Monjezi MR. Understanding the Role of Integrins in Breast Cancer Invasion, Metastasis, Angiogenesis, and Drug Resistance. Oncogene (2021) 40(6):1043–63. doi: 10.1038/s41388-020-01588-2 33420366

[42] Baxevanis CN Fortis SP Perez SA. The Balance Between Breast Cancer and the Immune System: Challenges for Prognosis and Clinical Benefit From Immunotherapies. Semin Cancer Biol (2021) 72:76–89. doi: 10.1016/j.semcancer.2019.12.018 31881337

[43] Place AE Jin Huh S Polyak K. The Microenvironment in Breast Cancer Progression: Biology and Implications for Treatment. Breast Cancer Res: BCR (2011) 13(6):227. doi: 10.1186/bcr2912 22078026 PMC3326543

