# Supplementary figures and images for: Expression Patterns of Ezrin and AJAP1 and Clinical Significance in Breast Cancer
Source: Front Oncol. 2022 Mar 4;12:831507. doi: 10.3389/fonc.2022.831507 (PMC8931223; doi:10.3389/fonc.2022.831507)

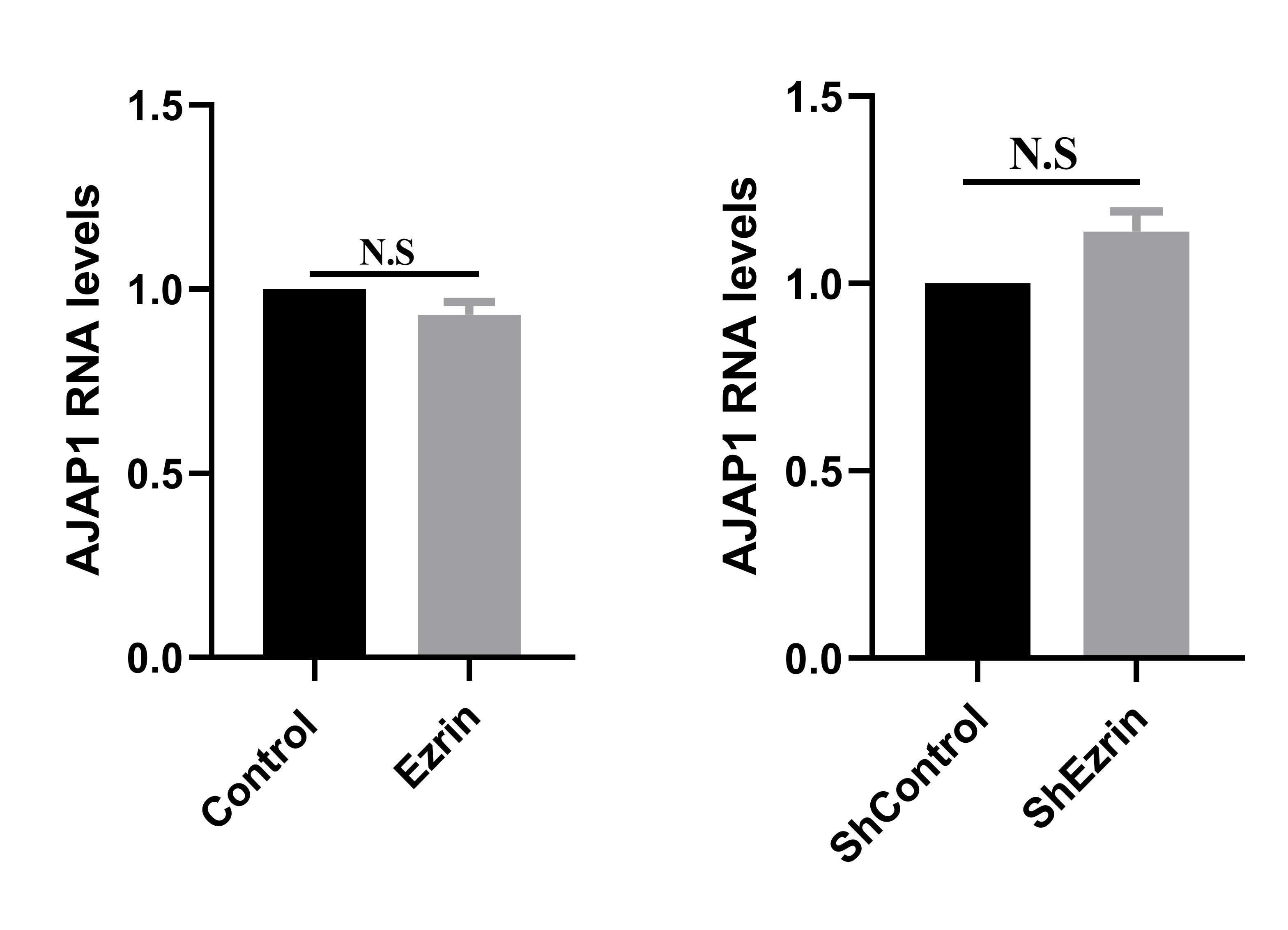

Supplement: Supplementary Figure 1 — qRT-PCR results of AJAP1 expression in Ezrin overexpressed MDA-MB-231 cells and Ezrin-silenced T47D cells. N.S., no significance. [file Image_1.tif]
